# Dual Modification of Porous Ca-P/PLA Composites with APTES and Alendronate Improves Their Mechanical Strength and Cytobiocompatibility towards Human Osteoblasts

**DOI:** 10.3390/ijms232214315

**Published:** 2022-11-18

**Authors:** Monika Biernat, Aleksandra Szwed-Georgiou, Karolina Rudnicka, Przemysław Płociński, Joanna Pagacz, Paulina Tymowicz-Grzyb, Anna Woźniak, Marcin Włodarczyk, Mateusz M. Urbaniak, Agnieszka Krupa, Paulina Rusek-Wala, Natalia Karska, Sylwia Rodziewicz-Motowidło

**Affiliations:** 1 Biomaterials Research Group, Łukasiewicz Research Network-Institute of Ceramics and Building Materials, Center of Ceramic and Concrete in Warsaw, Cementowa 8, 31-983 Kraków, Poland; 2 Department of Immunology and Infectious Biology, Faculty of Biology and Environmental Protection, University of Łódź, Banacha 12/16, 90-237 Łódź, Poland; 3 Bio-Med-Chem Doctoral School, University of Łódź and Łódź Institutes of the Polish Academy of Sciences, Banacha 12/16, 90-237 Łódź, Poland; 4 Faculty of Chemistry, University of Gdańsk, Wita-Stwosza 63, 80-308 Gdańsk, Poland

**Keywords:** biocomposites, PLA, hydroxyapatite, surface modification, mechanical strength, cytobiocompatibility, cell adhesion, osteoconduction, sodium alendronate

## Abstract

Synthetic implants are used to treat large bone defects that are often unable to regenerate, for example those caused by osteoporosis. It is necessary that the materials used to manufacture them are biocompatible and resorbable. Polymer-ceramic composites, such as those based on poly(L-lactide) (PLLA) and calcium phosphate ceramics (Ca-P), are often used for these purposes. In this study, we attempted to investigate an innovative strategy for two-step (dual) modification of composites and their components to improve the compatibility of composite components and the adhesion between PLA and Ca-P whiskers, and to increase the mechanical strength of the composite, as well as improve osteological bioactivity and prevent bone resorption in composites intended for bone regeneration. In the first step, Ca-P whiskers were modified with a saturated fatty acid namely, lauric acid (LA), or a silane coupling agent γ-aminopropyltriethoxysilane (APTES). Then, the composite, characterized by the best mechanical properties, was modified in the second stage of the work with an active chemical compound used in medicine as a first-line drug in osteoporosis—sodium alendronate, belonging to the group of bisphosphonates (BP). As a result of the research covered in this work, the composite modified with APTES and alendronate was found to be a promising candidate for future biomedical engineering applications.

## 1. Introduction

The clinical, safe and effective treatment of large bone defects (caused by tumor resection, trauma or congenital diseases) or the improvement of healing in fractures without or with delayed fusion continues to be a frequently discussed topic [1]. Approximately 4 million surgeries are performed annually worldwide using various methods, making bone the second most commonly transplanted tissue. One of the treatment options for such defects is the transplantation of synthetic bone graft substitutes [2]. Compared to the allograft method, synthetic products show better biocompatibility, lower risk of disease transmission and better acceptance among patients [2]. In order to be used as safe filling materials in injuries associated with the occurrence of bone defects, synthetic materials must meet a number of requirements. They should be biocompatible and resorbable. These materials should also induce regeneration process so that they are replaced with freshly synthesized bone tissue. Therefore, they should have an appropriate microstructure and optimal pore size that will allow good cell penetration, ingrowth of tissue, rapid vascularization and ease of nutrient delivery [3]. The mechanical strength of such filling implants should be suitable for the safe conduction of surgery and should withstand the mechanical load during movement [4]. The in vitro biocompatibility criteria that should be met by implant materials for applications in bone regenerative medicine also take into account the viability of cells in direct contact with the implant [5]. The biocompatibility of composites alone does not ensure the maintenance of the proper regeneration process and immune reactions, which are crucial for the healing process and directing tissue macrophages to the regulatory profile [6,7,8].

The ability to meet such diverse requirements is expected primarily from polymer-ceramic composite materials [9,10,11]. The biodegradable polymer poly(L-lactide) (PLLA) is especially widely used in orthopedic surgery and tissue engineering scaffolds [12,13]. As the PLLA is hydrophobic and its degradation causes the formation of acidic monomers capable of causing inflammatory and allergic reactions [14], calcium phosphate (Ca-P) ceramics, i.e., hydroxyapatite (HAp) or multiphasic Ca-P, are often added to the polymer matrix [15]. They improve osteoconductivity of composite and neutralize acidity catabolite of PLLA. The addition of Ca-P ceramic particles, especially in form of thin whiskers, is also a well-known method for the strengthening of polylactide composites [16,17].

The most sensitive part of a polymer-ceramic composite is the interfacial surface between the hydrophobic polymer and hydrophilic Ca-P particles. It is the first layer that undergoes degradation, which may cause the filler from the polymer matrix to detach easily, and thus reduce the mechanical strength of the implant [18]. Moreover, the incompatibilities of the two components and the inherent high surface energy of hydroxyapatite crystals favor their aggregation, which also leads to the development of poor mechanical properties.

The studies on overcoming these problems have focused on the modification of the hydroxyapatite surface through the application of various types of modifiers to increase the adhesion between the composite phases, and to impart electrostatic and steric stabilization to the filler particles. The use of surfactants and polyelectrolytes has been reported; e.g., silane coupling agents [16,19], polyacids [20], or saturated fatty acids [21]. A well-known solution is also the grafting of molecules that are miscible with polymers onto the filler particles [22,23,24]. Moreover, the development of bone implant biomaterials is constantly evolving in response to the needs of patients, and there are more and more studies related to biomaterials enriched with the active particles of drugs, growth factors or peptides [25,26,27].

In this study, we aimed to investigate a new strategy involving the two-stage (dual) modification of composites and composite components to obtain a porous biomaterial with improved physicochemical and biological properties.

The first stage of the work focused on the effect of the addition of multiphasic Ca-P whiskers on the composite microstructure, and on the influence of the surface modification of Ca-P whiskers on the mechanical and biological properties of porous Ca-P/PLA composites. The aim of this part of our study was to improve the mutual compatibility and adhesion between composite components: Ca-P whiskers and PLA matrix. We investigate and compare the effect of two types of modifiers: a saturated fatty acid in the form of lauric acid (LA) and a silane coupling agent in the form of γ-aminopropyltriethoxysilane (APTES), which vary in the chemical structure and biological properties.

Since the main challenge for biomaterials is their complete biocompatibility and lack of cytotoxic effect, as the first modifier for Ca-P whiskers, we chose the readily available, natural saturated fatty acid found in breast milk—lauric acid [28]. It is considered to be non-toxic and safe, both internally and externally, and moreover, it shows activity against harmful Gram-positive bacteria: *P. acnes, S. aureus* and *S. epidermidis* [29] and inhibits the growth of Gram-negative bacteria [30]. Another important feature in the use of LA in biodegradable composites is that it is biodegradable.

The second selected modifier—APTES is a popular silane coupling agent suitable as a surface modifier for glass and ceramic particles [31,32]. Silanes stimulate adhesion and can act as bridging agents for both inorganic and organic materials. Moreover, silanes appropriately anchored on HAp remain non-cytotoxic [19,33]. There are several studies on the use of APTES as a modifier to improve the strength properties of biodegradable composites, as well as to improve the adhesion of the material to tissues [16,34].

The innovation of our study is a new strategy of dual modification of the composites. The first-stage obtained composite including surface modified Ca-P whiskers, characterized with the best mechanical properties, was modified in the second step of the work with an active chemical compound used in medicine as a first-line drug for osteoporosis—sodium alendronate, which belongs to the bisphosphonates (BP) group. The mechanism of action of this drug is based on its binding to hydroxyapatites in the bones and its inhibiting the activity of osteoclasts, which cause bone tissue resorption [35,36]. It does not inhibit the bone formation process, and thus causes a gradual increase in bone mass. As the BPs show extremely low bioavailability, poor gastrointestinal absorption and a number of adverse effects when administered orally [37,38], there are known examples of studies developing new methods of local delivering of bisphosphonates to the body, e.g., in the form of drug-eluting implants [39].

To the best of our knowledge, there are no reports in the literature on the combination of two types of modification at two different stages of composite formation: (1) surface modification of multiphasic Ca-P particles used as a reinforcing composite filler, and (2) surface modification of the Ca-P/PLA porous composite. The goals of the dual modification strategy were also to improve the compatibility of the composite components, improve the adhesion between the PLA and Ca-P whiskers, and increase the mechanical strength of the composite, with the aim of improving the osteological bioactivity and prevention of bone resorption in composites dedicated for bone regeneration.

## 2. Results and Discussion

### 2.1. Properties of the Ca-P Whiskers and Their Surface Modification

**Figure 1:**
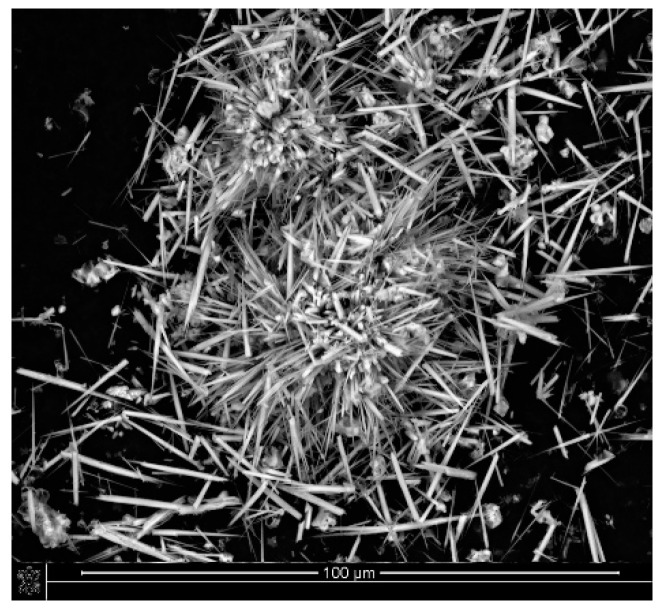
SEM image of Ca−P whiskers.

The Ca-P whiskers used in the work as composite fillers were obtained as in our previous work and were characterized by XRD and FTIR analysis. As we showed in previous work, they revealed a triphasic product containing hydroxyapatite, whitlockite and calcium pyrophosphate in amounts of approximately 72%, 15% and 12%, respectively [40]. The phase composition of the whiskers makes them a promising material for applications in bone-regenerative medicine. The high content of hydroxyapatite can provide bioactivity and osteoconductivity in the final biocomposites [41], and the presence of whitlockite and calcium pyrophosphate may increase the ability to create a permanent and strong connection between the implant and the bone [42,43,44]. According to literature data, they show also a faster degradation rate than the neat hydroxyapatite, which is considered as non-biodegradable, which indicates the possibility of obtaining a biomaterial with a controlled resorption rate [45,46,47]. A SEM image of the obtained whiskers is shown in Figure 1. The mean length of the whiskers was 18.84 ± 4.06 μm, and their mean width was 1.13 ± 0.24 μm. According to Mallick [48], the ratio of length-to-diameter of the whiskers plays an important role in composites supported with fibrous materials. It is assumed that the greater this ratio, the greater the fiber’s ability to reinforce the composite. The length-to-diameter ratio of the obtained whiskers equaled approximately 16.7; thus, they are a promising reinforcing material in PLA composites.

**Figure 2:**
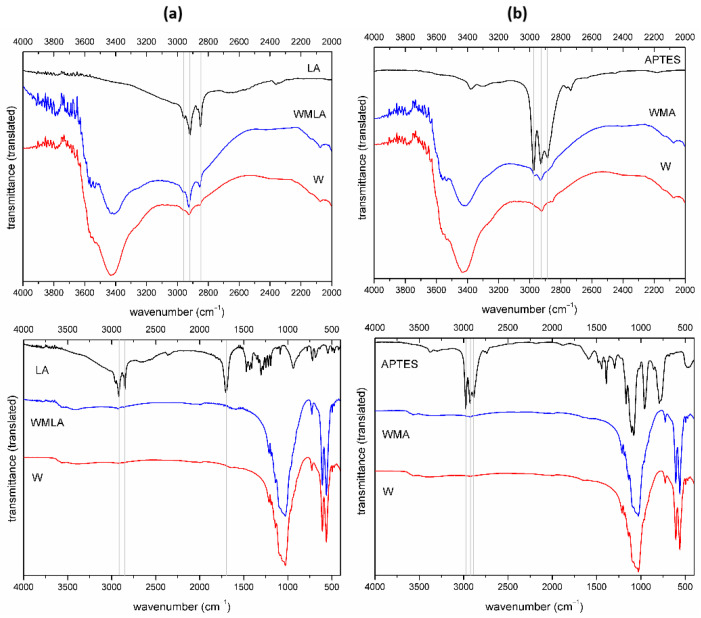
FTIR spectrum of: (**a**) Ca−P whiskers as received, LA and LA−modified whiskers; (**b**) Ca−P whiskers as received, APTES and APTES−modified whiskers (different wavenumbers range).

To further improve the mechanical strength of the composites, we decided to modify the whiskers with LA or APTES. Ca-P whiskers modification was confirmed by FTIR, TGA and STEM observation. The FTIR spectra of raw whiskers and modified materials (LA-whiskers (WMLA) and APTES- whiskers (WMA)) are shown in Figure 2.

In the spectrum of LA-modified Ca-P whiskers (WMLA), there are characteristic bands of hydroxyapatite, whitlockite and calcium pyrophosphate [49,50,51]. The band at 2964 cm^−1^ can be assigned to anti-symmetric C-H stretching vibration in CH_3_ group, the bands at 2852 cm^−1^ and 2919 cm^−1^ are assigned to symmetric and asymmetric stretching C-H vibration in CH_2_ groups, respectively. The difference for modified whiskers is due to the intensity of the bands at 2926 cm^−1^ and 2855 cm^−1^ highlighted in the figure (Figure 2a).

APTES-modified Ca-P whiskers (WMA) similarly to the above showed no additional bands derived from the modifier but the higher intensity of CH_2_ and CH_3_ bands at 2969 cm^−1^ and 2926 cm^−1^ (Figure 2b) was observed. The most characteristic bands of APTES modifier at approximately 1000 cm^−1^ (1167/1104/1080 cm^−1^) are invisible probably due to the overlapping with bands originating from the whiskers.

**Figure 3:**
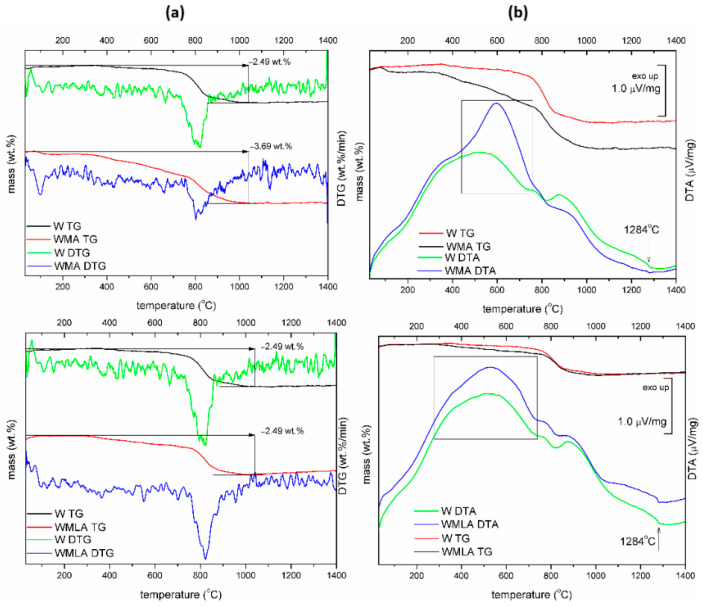
TG−DTG curves (**a**) and TG−DTA curves (**b**) for Ca−P whiskers unmodified and modified with LA and APTES.

The modifier content on the Ca-P whiskers’ surfaces was quantitatively determined from the TGA curves presented in Figure 3. The curves of the unmodified whiskers usually consisted of up to four mass loss regions up to 500 °C. At first (50–150 °C), the evaporation of physically adsorbed water on the whiskers’ surfaces was observed, and the second region (150–500 °C) corresponded to the evaporation of chemically adsorbed water, crystal water and lattice water. In our experiments, whiskers showed initial mass loss of about 0.17 wt.%; this was related to lattice water lost (200–450 °C), which was impossible to separate from the consequent dehydroxylation of surface phosphorous (450–730 °C). In the range of 730–1020 °C, dehydroxylation of hydroxyapatite can be observed [52]. The curves of LA-modified whiskers consisted of additional mass loss that corresponded to the oxidation of organic matter. A similar observation was made by other researchers for β-TCP modified with stearic acid [21,53]. The curves of APTES-modified whiskers consisted of three mass loss regions, which corresponded to lattice water lost (200–450 °C); dehydroxylation of surface phosphorous vs. APTES decomposition (450–730 °C); and dehydroxylation of hydroxyapatite (730–1020 °C). Moreover, the DTA curve showed the prominent exothermal effect between 440–740 °C, most probably related to the amine group decomposition during heating.

The approximate modifier amount was calculated from the difference in mass loss at 730 °C of unmodified and surface-modified Ca-P whiskers. The mass loss at 730 °C was 0.19 wt.% for unmodified Ca-P whiskers and 0.48 wt.% and 1.6 wt.% for LA-modified Ca-P whiskers and APTES-modified Ca-P whiskers, respectively. Thus, the content of LA on the whiskers’ surfaces reached 0.29 wt.%, and the content of APTES on the whiskers’ surfaces reached 1.41 wt.%.

**Figure 4:**
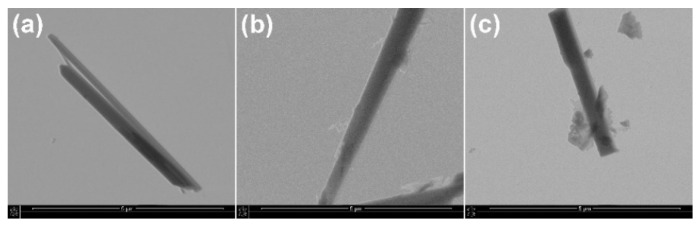
STEM images of (**a**) Ca−P whiskers as received, (**b**) APTES−modified Ca−P whiskers; (**c**) LA−modified Ca−P whiskers.

The surface modifications with lauric acid and with γ-aminopropyltriethoxysilane were also confirmed by observations on STEM micrographs (Figure 4). The smooth surface and clear contours of the Ca-P whiskers became rough and indistinct after modification. It can be seen that there are small fragments of modifier on the surfaces of the whiskers.

### 2.2. Properties of the Porous Composites with the Addition of Unmodified and Modified Ca-P Whiskers

#### 2.2.1. Microstructure

**Figure 5:**
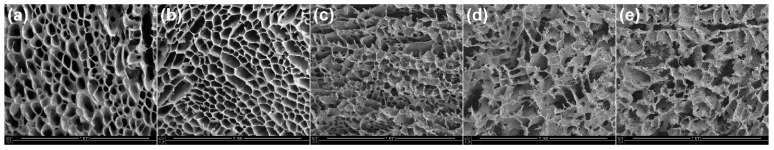
Dependence of the microstructure of porous composites of different Ca−P whisker content in composites: (**a**) 5PLA; (**b**) 5PLA_10W; (**c**) 5PLA_20W; (**d**) 5PLA_30W; (**e**) 5PLA_50W.

The microstructure of the obtained composites was investigated by SEM, which proved that all the composites were characterized by porous structure with open porosity. Manipulation of the number of whiskers obtained porous scaffolds with different pore sizes of 23–405 μm (Figure 5). The observed microstructure and the pore sizes were suitable to permit good cell penetration, ingrowth of tissue, rapid vascularization and ease of nutrient delivery [3].

With a moderate addition of Ca-P whiskers (10 wt.%), the microstructure of the composites did not change in a significant way; however, more regular pores were observed. This was possibly due to the Ca-P particles’ action as nucleation agents during the freezing in the lyophilization process, which resulted in the formation of a larger number of slightly smaller pores. The share of Ca-P whiskers in the amount of 20 wt.% and higher resulted in the formation of composites with disturbed microstructure, in which pores were larger but difficult to distinguish individually.

**Figure 6:**
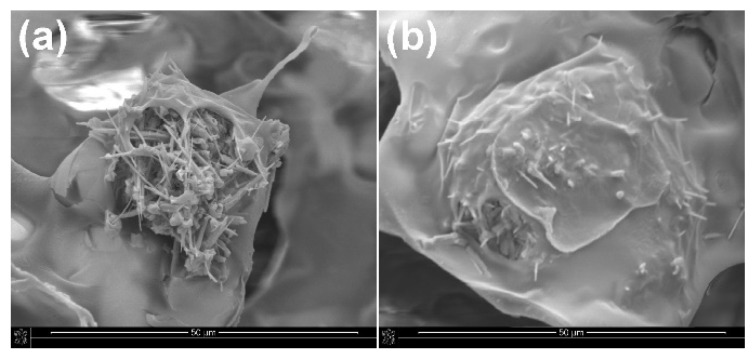
SEM images of porous composites with the addition of (**a**) unmodified and (**b**) APTES−modified Ca−P whiskers.

Ca-P whiskers in the porous structure of the obtained composites were located or anchored in the thin walls between the pores. In composites with the addition of unmodified whiskers, the whiskers in some places of the porous structure protruded from the walls of the scaffold, while in composites with the addition of modified whiskers, the whiskers were more well-covered with the polymer matrix (Figure 6). The chemical compatibility of the surface between the whiskers and the polymer matrix was increased by the modification process of the whiskers. This may improve the mechanical properties of porous composites.

#### 2.2.2. Mechanical Properties

**Figure 7:**
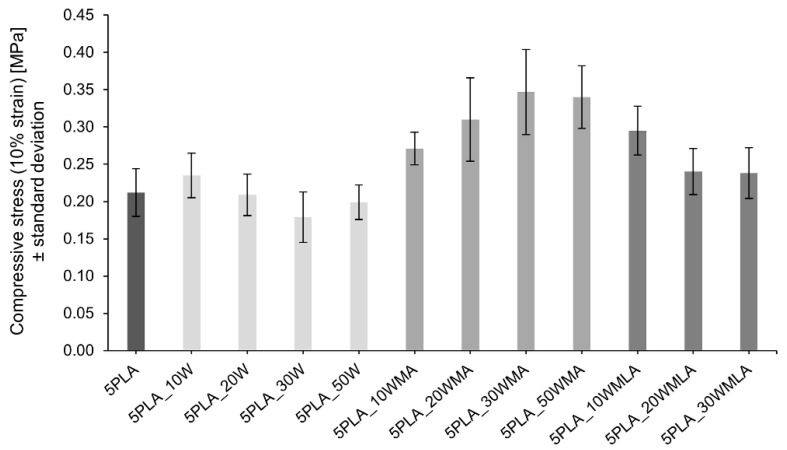
Influence of Ca−P whisker addition and its modification on the compressive strength (10% strain) of composites based on 5% polylactide solution.

The obtained Ca-P whiskers were applied in porous composites as a reinforcing filler. The mechanical strength of the composites was determined by compressive strength tests. Figure 7 shows the effect of the addition of various amounts of Ca-P filler and the effect of its modification on the mechanical strength (compressive stress at 10% strain) of composites based on 5% PLA solution.

The addition of a moderate amount (10 wt.%) of unmodified whiskers caused an increase in the compressive strength of the porous composites from 0.212 ± 0.032 MPa up to 0.235 ± 0.030 MPa. The further increase in the whisker amount in the composite led to a slight depression in the compressive strength. A similar trend was observed for materials with LA-modified whiskers; however, the initial value of the compressive strength for 5PLA_10WMLA was higher than for composites with unmodified whiskers, and reached 0.295 ± 0.033 MPa.

For composites with the addition of APTES-modified whiskers, the mechanical compressive strength increased up to 30 wt.% of the proportion of whiskers in the composite (0.271 ± 0.022 MPa) and only after this limit was exceeded did it begin to decrease. The observed dependencies are identical for all the compressive stress results determined at the strains from 10%–50% (Table 1).

### 2.3. Modification of Ca-P-Reinforced Composite with Sodium Alendronate

The composite with the addition of 30 wt.% APTES-modified whiskers, the compressive strength of which was the highest among the tested composites, was next surface-modified with sodium alendronate. This dual modification was aimed at obtaining a mechanically strengthened material with good biological properties at the same time as the action of sodium alendronate that binding to hydroxyapatites in the bones and inhibiting the activity of osteoclasts, which cause bone tissue resorption [35,36].

**Figure 8:**
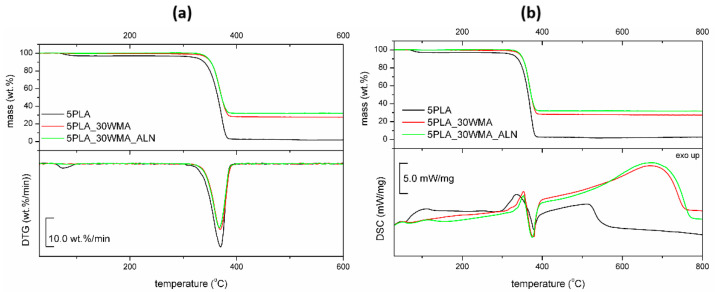
TG−DTG curves (**a**) and TG−DSC curves (**b**) for porous composites based on 5% PLA suspensions and its composites.

The obtained “dual-modified” composite was characterized by FTIR and thermal analysis to confirm the modification. Simultaneous TG-DSC thermal investigation (Figure 8) on composites showed that they differed slightly in their thermal stability—the lowest was observed for 5PLA. The solid residue after TG-DSC measurements was related to the filler addition and was close to that reported in the preparation procedure. All materials showed initial mass loss due to humidity evolution (30–140 °C) and one-step decomposition in the range of 300–400 °C. The highest initial mass loss of 2.9 wt.% was registered for 5PLA. Dual-modified material showed additional mass loss (0.42 wt.%) at 305 °C that may be related to the decomposition amine groups in the ALN structure, as reported previously [54].

DSC analysis revealed initial loss of water (till 100 °C) and heat effects during the degradation process (300–800 °C). The degradation of the dual-modified composite was characterized by a 27% higher heat effect, which may be considered to have proved the modification. The possible partial melting of the polymer was not observed.

**Figure 9:**
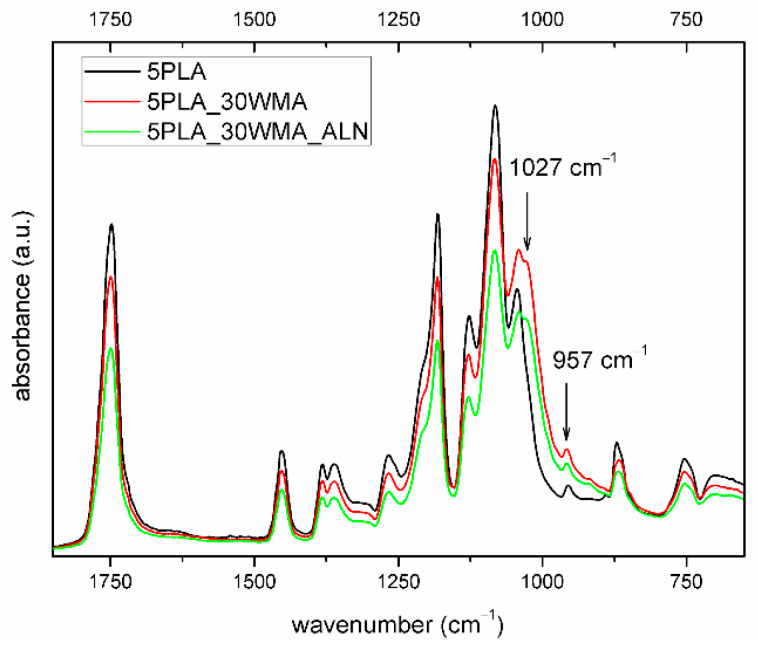
FTIR spectrum of porous composites based on 5% PLA suspensions and its composites (2000–600 cm^−1^).

No particular differences were observed between the composites in their structure as analyzed by IR spectroscopy (Figure 9). However, the group of signals in the range of 1060–970 cm^−1^ varied in their intensity, width and position. For the composites, a new band at 1027 cm^−1^ was registered that could be assigned to the C-O-C band vibration and the P-O and Si-O bonds, which were all observed in the modifiers’ structures. The band at 957 cm^−1^ identified for all composites was assigned to the amorphous part of the polymer, and corresponded to CH_3_ deformation vibrations [55].

### 2.4. Properties of Dual-Modified Porous Composite

#### 2.4.1. Microstructure and Mechanical Properties

**Figure 10:**
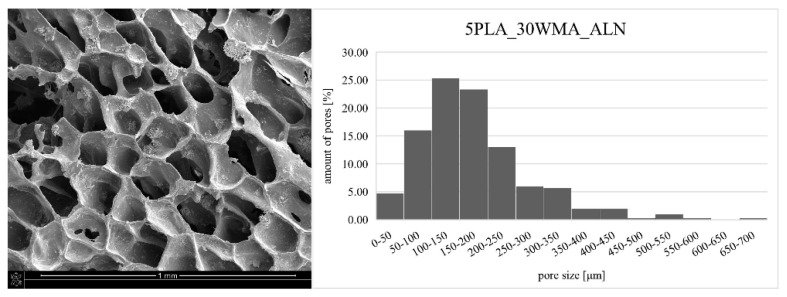
SEM image of dual-modified composite: 5PLA_30WMA_ALN (**left**) and pore size distribution of this composite (**right**).

The composite obtained by the dual modification route was subjected to microstructure and mechanical tests. The SEM image of the 5PLA_30WMA_ALN composite (Figure 10) confirms its porous structure and open porosity. ALN modification did not change the pore sizes of the composite, which was in the range of 24–679 μm. Simultaneously, the mechanical strength of the ALN-modified composite stayed close to the values determined for the composite without ALN. The compression strength of the “dual-modified” composite at 10% strain equaled 0.247 ± 0.027 MPa (Table 2). This value was higher than the value of the compression strength for composite 5PLA in this research work, and was higher than the value of compression strength for the composite 5% PLLA obtained by other researchers (0.13 ± 0.01 MPa for 10% strain) [56]. It was similar to strength of the composites made of the same polymer but with other HAp-based fillers, which had compression strength values of about 0.2–0.3 MPa [57]. It also had better strength than the composites HAp/β-TCP obtained by the freeze-casting method, which showed a strength of about 0.54 MPa, while our composite showed a strength of 0.653 ± 0.042 MPa (for 50% strain) [58]. The composite obtained by double modification had a strength within the range of values obtained for natural cancellous bone (compressive strength of 0.1–30 MPa) [59].

Pore size affects both the strength of the composite (the larger the pores, the lower the strength) and the biological properties. Large pore sizes (400–600 μm) can promote vascularization and osteogenesis in vivo, while small pore sizes (around 100 μm) can contribute to early cell adhesion in vitro [2,60]. Hence, an ideal scaffold with desirable mechanical and biological properties should exhibit a diverse pore size distribution. Composite 5PLA_30WMA_ALN had good pore size distribution (Figure 10). Pores in the range of 100–150 µm were the most common pores in the composite, but pores with sizes above 300 µm also occurred. The average pore size in the composite was 177.05 µm.

#### 2.4.2. Sodium Alendronate Release

**Figure 11:**
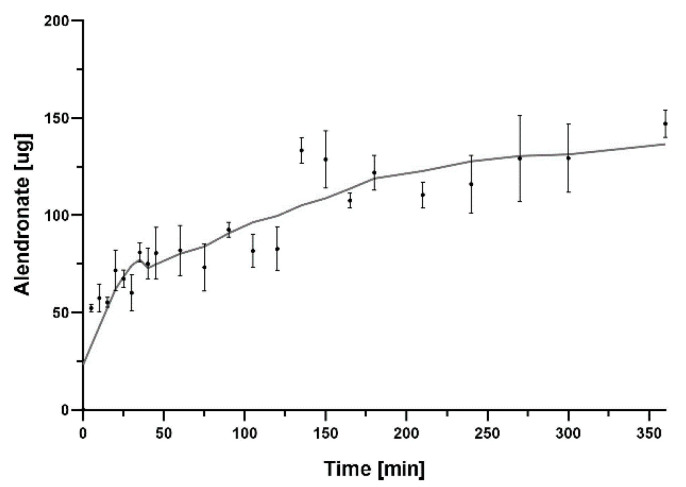
Graph showing sodium alendronate release from Ca−P/PLA scaffold over 360 min. The mass of sodium alendronate released was converted into a 50 mg disc of composite. The data are presented as the mean ± SD for the three separate experiments.

In this study, we incorporated sodium alendronate, a drug for osteoporosis, into a porous Ca-P/PLA composite scaffold. The last step in the scaffold preparation process included the adsorption of sodium alendronate. In order to determine the kinetics of the release of this drug from the scaffold, the process was measured. Sodium alendronate incorporated in composite showed a constant release from the scaffold over the 200 min. Then, after 300 min, the level of sodium alendronate remained almost unchanged. The kinetics profile of sodium alendronate release is presented in Figure 11. The HPLC results show that the sodium alendronate was adsorbed onto the Ca-P/PLA scaffold.

### 2.5. Biological Properties

#### 2.5.1. Cytobiocompatibility of Scaffolds

**Figure 12:**
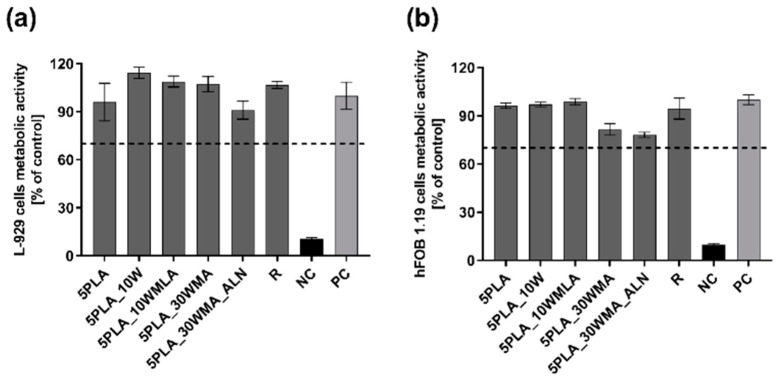
(**a**,**b**). The metabolic activity of mouse L929 fibroblasts (**a**) and human hFOB 1.19 fetal osteoblasts (**b**) after 24 h incubation with composites (5PLA, 5PLA_10W, 5PLA_10WMLA, 5PLA_30WMA, 5PLA_30WMA_ALN). PC—positive control of L929 (**a**) or hFOB 1.19 (**b**) metabolic activity; NC—negative control of L929 (**a**) or hFOB 1.19 (**b**) metabolic activity (cell cultures treated with 2% saponin solution); R—biomaterial derived from medically certified peripheral venous catheter. The data are presented as the mean ± SEM for the four separate experiments.

We investigated the effect of the modification of Ca-P/PLA whiskers with LA and APTES on the metabolic activity of L929 fibroblasts and hFOB 1.19 osteoblast. We showed that none of the PLA- based composites, the composites modified with multiphasic Ca-P whiskers (5PLA10W: 103.1% ± 13.0), the whiskers modified with LA (5PLA10WMLA: 94.5% ± 17.3) and APTES (5PLA30WMA: 81.8% ± 11.2%) or the dual-modified composites (5PLA30WMA_ALN: 94.1% ± 12.8%) did not caused a notable decrease in the metabolic activity of standard L929 fibroblast, and these composites were thus confirmed to be cytobiocompatible in vitro (Figure 12a).

Similarly, the tested composites were cytobiocompatible with human fetal osteoblasts hFOB 1.19 (5PLA: 85.6% ± 11.5; 5PLA10W: 85.7% ± 11.0; 5PLA10WMLA: 79.5% ± 10.6%; 5PLA30WMA: 77.5% ± 10.9%; 5PLA30WMA_ALN: 80.3% ± 10.3%) (Figure 12b). In summary, we showed that modifications of the Ca-P/PLA scaffolds did not affect the viability of L929 fibroblasts, a finding that meets the ISO in vitro criteria of biocompatibility and is consistent with the findings of other authors, who had investigated the influence of titanium screws coated with PLA and modified with ZnO and SiO_2_ on L929 cell viability, and had shown that those materials were cytobiocompatible [61]. In addition, the scaffolds remained cytobiocompatible towards osteoblasts—cells that stay in direct contact with a Ca-P/PLA-based composite intended for bone regeneration.

#### 2.5.2. Immunocompatibility of Biomaterials with Human Monocytes

**Figure 13:**
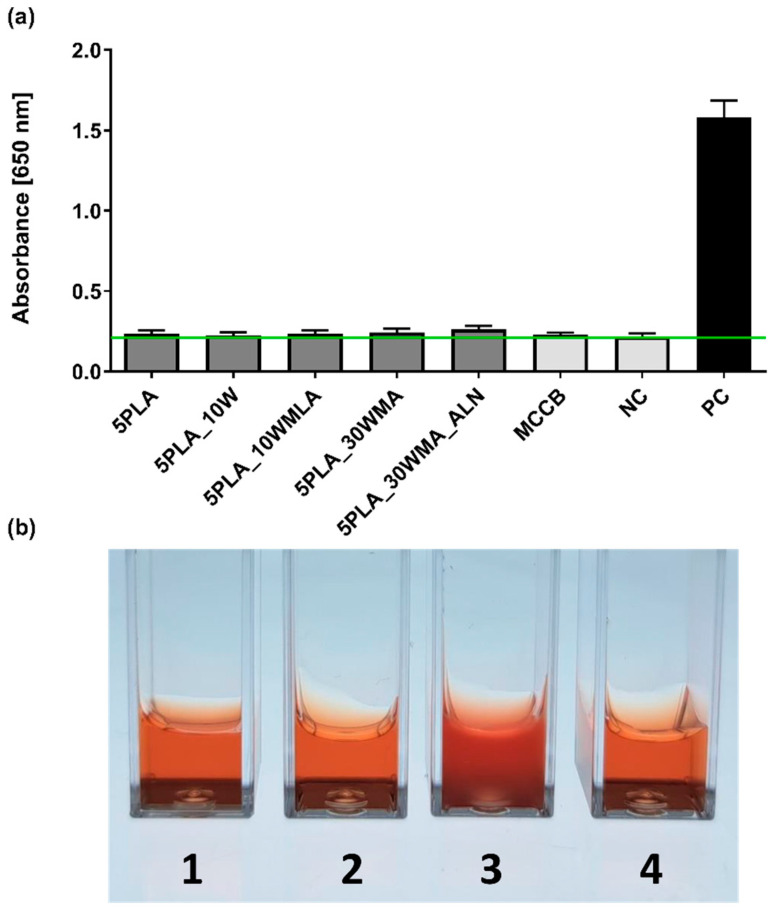
(**a**) The NF-κB induction in human THP1-Blue™ NF-ĸB monocytes after 24 h incubation with composites (5PLA, 5PLA_10W, 5PLA_10WMLA, 5PLA_30WMA, 5PLA_30WMA_ALN). Negative controls of the monocyte’s activation—the cells incubated without composites (NC) and stimulated with medically certified control biomaterial (MCCB); positive control—monocytes stimulated with *E. coli* LPS (PC). The data are presented as the mean ± SD for the four separate experiments. The green line indicates the physiological level (0.24 ± 0.02) of the non-stimulated monocytes. (**b**) Complement fixation test—lysis of erythrocytes: tube 1 (5PLA_30WMA), tube 2 (5PLA_30WMA_ALN), tube 4 (positive control) compared to tube 3 (negative control, no hemolysis).

The human monocytic cell line THP1-Blue™ NF-ĸB was used to assess the level of NF-ĸB activation resulting from Ca-P/PLA composites and their modifications. The in vitro reporter monocytes are the indicator of NF-κB transcription factor activation detected indirectly by the quantification of SEAP release to the cell culture medium. The activation is mediated by the interaction of the ligand (present in the sample, in this case possible LPS contamination) with TLR receptors (Figure 13a). THP1 -Blue™ NF-ĸB activation tests were augmented with a complement fixation test (Figure 13b) to exclude complement-mediated immune responses towards the tested composites.

We showed that the tested samples did not activate human monocytes, and they exhibited a resting state similar to the untreated controls (Figure 13). The analysis of variance (ANOVA) with Tukey’s post-hoc test of the examined biomaterials (5PLA, 5PLA_10W, 5PLA_10WMLA, 5PLA_30WMA, 5PLA_30WMA_ALN) showed no differences between the levels of NF-κB activation (0.24 ± 0.02; 0.23 ± 0.02; 0.24 ± 0.02; 0.24 ± 0.02; 0.26 ± 0.02, respectively) compared to those in the unstimulated monocytes (NC: 0.22 ± 0.02) and cells exposed to certified control biomaterial approved for medical use (MCCB: 0.23 ± 0.01). By contrast, the monocytes stimulated with LPS of *E. coli* were highly activated (PC: 1.58 ± 0.08), which was manifested by the transcription of NF-kB nuclear factor and the intense release of alkaline phosphatase, when compared to untreated monocytes (NC: 0.22 ± 0.02). The obtained results suggest that the tested materials did not contain bacterial contaminants and that the structure of the material did not mechanically induce their activation.

The NF-κB signaling pathway is involved in the control of bone metabolism by its promotion of osteoclast differentiation and bone lysis processes. Activation of the NF-κB pathway leads to an increase in the number of active osteoclasts while reducing the metabolic activity of osteoblasts. NF-κB activation stimulates the release of inflammatory cytokines, such as IL-6 and TNF-α [62,63]. Controlling the activation of the NF-κB pathway is one of the essential purposes of the guided remodeling of implantable bone tissue. Inoue et al. reported that alendronate inhibited the PI3K–Akt–NF-κB pathway in the osteosarcoma cell line MG-63 [64]. The lack of activation of this signaling pathway seen for sodium alendronate-modified composites in our study could indicate similar effects, or may simply exclude the contamination of tested materials with pyrogenic substances.

To further evaluate the immunocompatibility of biomaterials with human cells and proteins, we performed a whole blood assay including complement-mediated immune response according to ISO 10993-4:2017 (Biological evaluation of medical devices—Part 4: Selection of tests for interactions with blood). Thus, the composites 5PLA_30WMA and 5PLA_30WMA_ALN were firstly incubated with standardized heat-inactivated human serum and fresh guinea pig serum (the source of complement) and then subjected to complement fixation text. As shown in Figure 13b, neither tested composites caused complement fixation; therefore, the hemolysis of sheep red blood cells (SRBC), which had been pre-bound to anti-SRBC antibodies, was observed. The acquired results additionally confirm the safety of the tested composites.

#### 2.5.3. Visualization of Cell Adhesion

**Figure 14:**
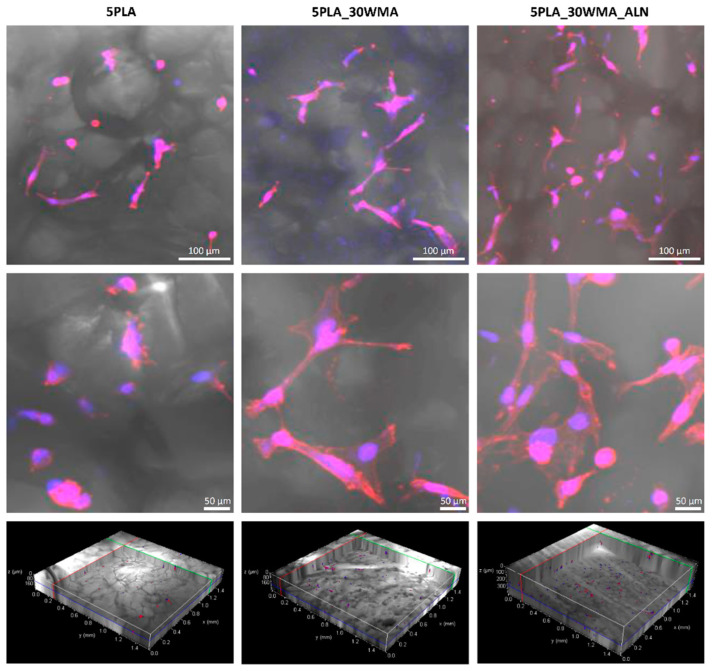
hFOB 1.19 colonization of composites (5PLA, 5PLA_30WMA and 5PLA_30WMA_ALN) after 24 h. The nuclei were stained with 300 nM 2-(4-amidinophenyl)-1Hindole-6-carboxamidine (DAPI), and Texas Red™ Phalloidin was used for F-actin staining (cytoskeleton). Samples were imaged with the wavelength values of 405 nm (excitation) and 430–480 nm (emission) for DAPI and with 595 nm (excitation) and 610–630 nm (emission) for Texas Red™ Phalloidin. Leica Application Suite X (LAS X; Leica Microsystems) was used for cell imaging. Each panel represents 2D pictures of foam scaffolds.

In this work, we also evaluated the hFOB 1.19 osteoblast adhesion on the surfaces of the materials 5PLA, 5PLA_30WMA and 5PLA_30WMA_ALN (Figure 14). We showed that osteoblasts could successfully colonize tested composites. Compared to 5PLA and 5PLA_30WMA foams, 5PLA_30WMA_ALN scaffolds displayed increased cell adhesion. While osteoblasts seeded on 5PLA composites had more oval shapes, the modification of biomaterials containing alendronate resulted in osteoblasts with elongated bodies. Moreover, no signs of physiological changes (e.g., apoptosis) were noted.

#### 2.5.4. Osteoconductive Properties

In the current study, we evaluated the level of osteocalcin, alkaline phosphatase, interleukin-6 and cells proliferation in cell lysates and supernatants obtained from composites colonized by hFOB 1.19 on days 7, 14, 21 and 28 and cultured in an osteoinductive environment at 39 °C. Our results indicate that the presence of alendronate significantly increases the osteoconductive potential of 5PLA_30WMA composites. We observed that the composite containing alendronate increased the osteocalcin (OC) production as well as the alkaline phosphatase (ALP) activity. These biomarkers of bone metabolism are indicative of bone turnover, including bone formation, mineralization [65] and the formation of hard tissue [66]. Moreover, the biomaterials modified with alendronate promoted cell proliferation. This observation may suggest that sodium alendronate on the surface of the composite supports osteoblastic cell growth and adhesion as well as differentiation, which are important aspects of bone regeneration.

**Figure 15:**
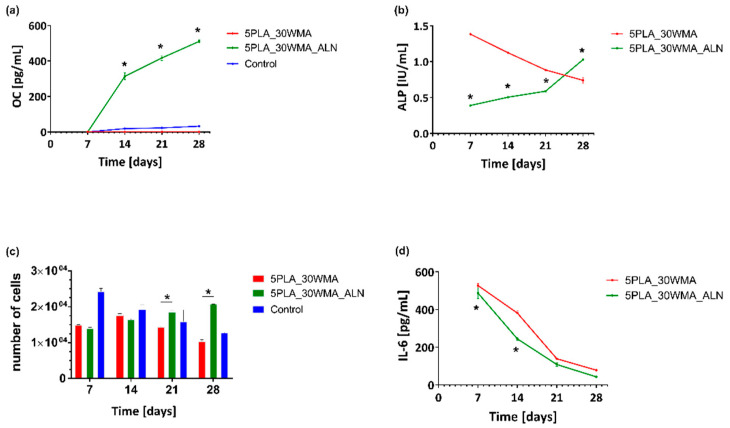
Analysis of (**a**) -osteocalcin (OC); (**b**) -alkaline phosphatase (ALP); (**c**) -proliferation; (**d**) -interleukin-6 in the lysates and supernatants obtained from hFOB1.19 cells cultured in the osteoinductive environment at 39 °C. The cells seeded on a non-osteoinductive surface (cell culture plate) were used as control for osteocalcin production (**a**) and proliferation (**c**). The results represent the mean ± SEM. * *p* < 0.05 between the 5PLA_30WMA and 5PLA_30WMA_ALN composites, based on the results of one-way ANOVA (Kruskal–Wallis test) evaluation.

The level of OC produced by hFOB 1.19 on the 5PLA_WMA composites fell below the minimum detectable level throughout the entire time course of the experiment (Figure 15a). Notably, the osteoblasts cultured in the presence of 5PLA_30WMA scaffold did not produce OC at any time point, whereas the level of this osteoinduction marker gradually increased in time in response to the 5PLA_30WMA_ALN composite, from 0.0 ± 0.0 pg/mL on day 7 to 510.6 ± 9.2 pg/mL on day 28. Similarly, the 5PLA_30WMA_ALN-driven OC production was accompanied by an increase in ALP activity over time, from 0.4 ± 0.001 IU/mL on day 7 to 1.4 ± 0.003 IU/mL on day 28 (Figure 15b).

It has been shown by Boanini et al. that MG63 osteoblasts cultured on samples containing a relatively high content of alendronate display an increased production of ALP and OC with respect both to the control and to pure hydroxyapatite [67].

In bone tissue regeneration, initial cell adhesion on the scaffolds is one of the critical factors resulting in cell differentiation. Komatsu et al. observed that local application of alendronate by calcium phosphate scaffolds enhances osteoblast differentiation and mineralization in vivo [68]. Moreover, the adhesion process of MG-63 osteoblasts was positively correlated with an increase in the amount of ALN coating the biphasic calcium phosphate scaffolds [69]. In the present study, to evaluate cell proliferation on PLA and PLA-modified composites, hFOB 1.19 were cultured in the milieu of the scaffolds. We showed that under prolonged culture, osteoblasts cultured in the milieu of an ALN-modified composite proliferated more efficiently than cells exposed to a control composite (5PLA_30WMA). The mean number of cells (MNoC) identified within 5PLA_30WMA_ALN scaffolds was significantly (*p* < 0.05) higher as compared to the proliferation rate in the presence of 5PLA_30WMA composite on days 21 (1.9 × 104 ± 433.0 and 1.4 × 104 ± 288.7, *p* < 0.05, respectively) and 28 (2.1 × 104 ± 57.7 and 1.0 × 104 ± 516.5, *p* < 0.05, respectively) (Figure 15c).

It is worth noting that the first physiological phase of the bone tissue regeneration process is inflammation, which requires the involvement of the pro-inflammatory cytokines. IL-6, among others, is known to be one of the strong proinflammatory cytokines, helping to control osteoblast differentiation and enhance the differentiation of osteoblast’s precursors. Moreover, IL-6 protects osteoblasts from apoptosis [70]. Analysis of the immunomodulatory cytokine IL-6 (Figure 15d) assayed in the supernatants of hFOB 1.19 cultured on PLA scaffolds under osteoinductive conditions (39 °C) showed that the secretion of IL-6 was similar for the cells grown on both composites, and the addition of alendronate had no effect on the release profile of IL-6 up to 28 days. The production of IL-6 decreased over time and reached 78.1 ± 3.5 pg/mL for 5PLA_30WMA and 42.9 ± 0.1 pg/mL for 5PLA_30WMA_ALN on day 28. The rapid release of IL-6 and the subsequent decrease in its production over time in the presence of these composites may support their positive role in the bone regeneration process. The presence of this cytokine is important to induce regenerative processes; however, the excessive production of this cytokine may initiate pathological chronic inflammation detrimental to the healing process.

**Figure 16:**
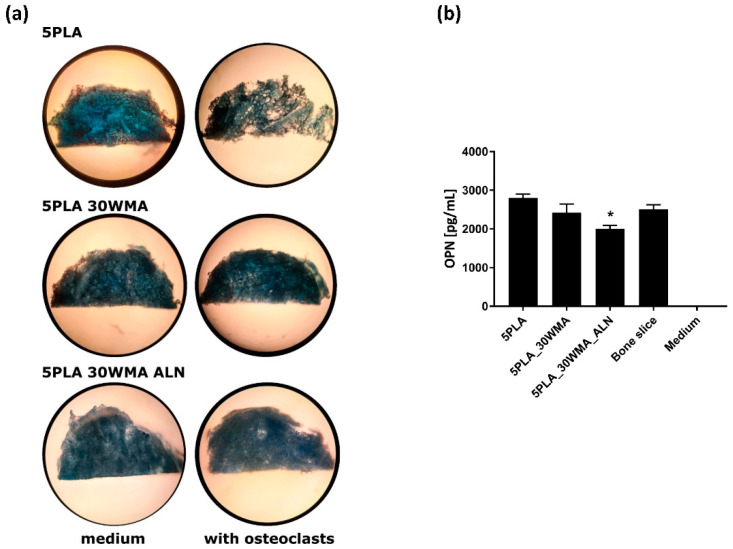
(**a**) Osteoclasts-mediated resorption of tested scaffolds, stained with Coomassie brilliant blue. (**b**) Analysis of the osteopontin (OPN) production in supernatants obtained from osteoclasts cultured at 37 °C on the corresponding scaffolds. The cells seeded on similarly sized bone slices were used as a control for osteopontin production. The results represent the mean ± SEM. * *p* < 0.05 between the 5PLA and 5PLA_30WAM_ALN composites, based on the results of one-way ANOVA (Tukey test) evaluation.

In order to evaluate the ability of our APTES- and sodium alendronate-modified materials to inhibit the activity of osteoclasts and thus prevent bone-like tissue material resorption, we carried out scaffold resorption assays, following the bone slice resorption protocol. We noticed that the PLA control material was sensitive to the activity of human monocyte-derived osteoclasts to a much higher degree than the APTES- and APTES/alendronate-containing materials (Figure 16a). Moreover, we evaluated the level of osteopontin produced by osteoclasts under each tested condition (Figure 16b). Our results indicated that the presence of alendronate significantly decreased the production of OPN as compared to the 5PLA control (2005 ± 90.5 pg/mL and 2804 ± 99 pg/mL, *p* = 0.004). OPN is one of the key components in osteoclast attachment to bone during resorption. Luukkonen et al. showed that osteoclasts secrete OPN into the resorption pits, where it may function as a chemokine for subsequent bone formation [71].

## 3. Materials and Methods

### 3.1. Materials

In order to obtain the Ca-P/PLA composites, the following components were used: starting calcium phosphate powder (β-TCP, 96%, Product No. 21218) supplied by Sigma-Aldrich (Poznań, Poland) as a product of Fluka Chemie GmbH, Buchs, Switzerland; 30% solution of H_2_O_2_ (Catalog No. BA5193111) supplied by Avantor Performance Materials Poland S.A., Gliwice, Poland; polylactide PLA-RESOMER^®^ LR 706 S (Evonik) dedicated for medical purposes (tissue engineering) characterized as poly(L-lactide-co-D,L-lactide) 70:30; 1,4-dioxane (Avantor) used as a solvent of polylactide; lauric acid (LA) (Sigma-Aldrich) and γ-aminopropyltriethoxysilane (APTES) (Sigma-Aldrich) used as surface a modifier for whiskers; sodium alendronate (ALN) (Sigma-Aldrich) as porous composite surface modifier; 99.8% ethanol (Avantor) and anhydrous toluene (Avantor).

### 3.2. Preparation and Surface Modification of Ca-P Whiskers

The short Ca-P whiskers were prepared in a one-pot synthesis, as with our previous work [40]. The β-TCP starting powder (4 g) was placed in a 250 mL capacity Pyrex glass bottle, and then 100 mL of 30% solution of H_2_O_2_ was added. The capped bottle was shaken for 2 min and heated for 48 h (undisturbed) in an electric oven in 95 °C. The whiskers obtained from the bottle were filtered, washed four times with 500 mL of distilled water and dried overnight at 90 °C.

The short Ca-P whiskers were next modified in two ways. (1) LA modification. The whiskers (6 g) were dispersed ultrasonically for 20 min in 20 mL of deionized water to form a suspension. Lauric acid (4.8 g) was dissolved in 10 mL of ethanol at 60 °C, poured into the whiskers suspension and mixed. The solution was stirred for 12 h at 120 R and 60 °C, and then the solvent was filtered out. The whiskers (WMLA) were washed three times with 10 mL of ethanol and dried at 60 °C for 24 h. (2) APTES modification. Before the modification, the whiskers were dried at 60 °C for 24 h to remove water from their surfaces. The whiskers (10 g) were added to 100 mL of APTES anhydrous toluene solution (0.1 mol/l), dispersed ultrasonically for 10 min and magnetically mixed for 48 h at 120 R and 37 °C under nitrogen atmosphere. Then, the whiskers (WMA) were centrifugally separated, washed four times with 40 mL of toluene and dried overnight at 50 °C.

### 3.3. Preparation and Surface Modification of Ca-P/PLA Porous Composites

Solutions of 5% polylactide were prepared by dissolving the polymer in 1,4-dioxane under stirring for 72 h. Then various amounts of Ca-P whiskers (modified: “WMA”—whiskers modified with APTES, “WMLA”—whiskers modified with lauric acid and “W”—unmodified whiskers) were added to the obtained solutions with fixed proportions of whiskers to polymer (Table 3).

The whiskers were suspended in the solutions with 10 min of ultrasonic dispersing and magnetic stirring until homogeneous. The obtained homogenous suspensions were frozen at −20 °C and were then transferred to the Christ BETA 1-16 lyophilizer. Porous composites were prepared by freeze-drying of frozen suspensions under −35 °C and a pressure of 0.06 MPa in the main drying stage and 0.005 MPa during the final stage for 48 h. The obtained composites were then washed several times with 1000 mL of deionized water and dried again by lyophilization according to the above procedure.

The selected Ca-P/PLA composite was next surface modified by the adsorption of sodium alendronate. The porous composite was dipped for 24 h in sodium alendronate solution (1 mg/mL) and then dried by lyophilization, obtaining “dual-modified composite” 5PLA_30WMA_ALN.

Additionally, prior to biological evaluation, the composites were sterilized by fast electron radiation at the Institute of Chemistry and Nuclear Technology (Warsaw, Poland). The set dose of radiation was 25 kGy, the speed of the transporter 0.462 m/min, and the set current 600 mA. This process was intended to destroy microbial contamination.

### 3.4. Characterization of the Obtained Materials

#### 3.4.1. X-ray Diffraction

The phase composition of Ca-P whiskers and modified Ca-P whiskers was analyzed by Bragg–Brentano X-ray diffraction method (XRD) on a Bruker-AXS D8 DAVINCI diffractometer designed for a copper anode tube (Bruker, Karlsruhe, Germany). The diffractograms were recorded in an angular range of 4° to 90° 2θ (Cu Kα), measuring step 0.019° and measurement time: 2 s/step. Quantitative analysis was performed by Rietveld method using the TOPAS v5 program.

#### 3.4.2. Fourier Transform Infrared Spectroscopy

A Fourier transform infrared spectroscopy (FTIR) was used to determine functional groups of whiskers, APTES-modified whiskers and LA-modified whiskers. The infrared spectra were recorded on a Bruker TENSOR 27 instrument equipped with a DLaTGS detector in transmission KBr mode. The analysis was performed in the wavelength range of 400 cm^−1^ to 4000 cm^−1^ with spectral resolution of 4 cm^−1^ and 64 scans. The baseline correction procedure was applied to the presented spectra. For PLA composites, FTIR-ATR mode was used to observe structural changes with a Platinum^®^ ATR accessory equipped in diamond crystal.

#### 3.4.3. Thermal Analysis

The thermal stability and behavior of powdered materials were analyzed with an STA F3 449 Jupiter Netzsch thermal analyzer. TG-DTA experiments were performed under dynamic flow of argon (70 mL∙min^−1^) to investigate the mass loss due the presence of modifier. Samples of about 10 mg were heated in an Al_2_O_3_ DTA pan with a lid and hole from 30 °C up to 1400 °C at a heating rate of 10 °C × min^−1^. The measurements were repeated twice for each sample. PLA composites were analyzed during TG-DSC measurements on STA F3 449 Jupiter^®^ Netzsch. For this purpose, a sample of about 4 mg was prepared and placed in an Al_2_O_3_ DSC pan with a lid and hole, and heated from 30 °C up to 1000 °C at a heating rate of 10 °C × min^−1^.

#### 3.4.4. Scanning Electron Microscopy (SEM/STEM)

The morphology of the whiskers was observed by field emission scanning electron microscope (SEM) (Nova NanoSEM 200, FEI). Imaging of sample microstructure was performed in high-vacuum conditions using an ETD detector at a 10 kV accelerating voltage. Before the study, the samples were covered with conductive material (10 nm gold film) using a Leica EM SCD500 sputter coater. The morphology of the whiskers before and after modification was also observed by a STEM detector installed in SEM. In STEM observations, the whiskers were placed on a copper mesh and observed at 25 kV accelerating voltage. The microstructure of composites was also observed by SEM. Whisker dimensions and composite macropore sizes were determined on the basis of microscopic images using measurement and annotation functions on the sample area. At least 50 measurements were performed.

#### 3.4.5. Compressive Strength

The mechanical properties of the porous composites were evaluated by compression tests. All samples were prepared as cylindrical specimens 1.2 (±0.007) cm length and 1.1 (±0.018) cm diameter. The compression tests were conducted on more than 5 specimens of each series at a cross-head speed of 1 mm/min on the Zwick Roell 5kN ProLine test machine. The stresses evident at the 10%, 20%, 50% compression levels were considered as indicating compressive strength.

#### 3.4.6. Sodium Alendronate Releasing

In order to establish a calibration curve for alendronate, three identical solutions of sodium alendronate were prepared, for which chromatographic analyses (HPLC) were performed. A standard alendronate solution (1 mg/mL) was prepared by dissolving the sodium alendronate in a water. Ten dilution points were designated. For the obtained results, the relationship between the area under the peak in the HPLC chromatogram and the concentration was determined using Shimadzu LC solution software (Shimadzu Co., Kyoto, Japan). The results of three separate measurements were averaged.

The composite foam (with sodium alendronate; without as a control) was weighed and placed in Eppendorf tubes, to which 1 mL of water was added. The composite foam was incubated at 37 °C, with gentle shaking (300 rpm) for 24 h. The sodium alendronate release from the composite was tested at the following time points: 0, 5, 10, 15, 20, 25, 30, 35, 40, 45, 60, 75, 90, 105, 120, 135, 150, 180, 240, 270, 300 and 360 min. The analysis of the released sodium alendronate was performed using a NEXERA X2 chromatography system (Shimadzu, Duisburg, Germany) consisting of two pumps (LC 30AD), a ELSD LTII detector, an SPD-M20A detector, an automatic dispenser (SIL 30AC), a column thermostat (CTO-20AC) and a degasser membrane (DGU-20A5R). HPLC analysis was performed with a linear 0–100% B gradient for 30 min on a bioZen™ (Phenomenex Inc., Torrance, CA, USA) 2.65 µm Glycan (2.1 mm × 250 mm) column, where A: 0.01 mM ammonium acetate and 0.1 mM acetic acid in an acetonitrile/water mixture of 7:3 (*v*/*v*); B: 0.01 mM ammonium acetate and 0.1 mM acetic acid in a acetonitrile/water mixture of 5:5 (*v*/*v*), pH 3.75. The volumetric flow rate was 0.25 mL/min. Peaks were detected on the ELSD detector. The injection volume was 10 µL for each sample. The mass of sodium alendronate released was calculated on the basis of the value of the peak area of the released sodium alendronate and the previously determined calibration curve for sodium alendronate.

### 3.5. Biological Properties

#### 3.5.1. In Vitro Cytocompatibility

Studies on cytotoxicity were carried out using murine fibroblasts (L929, Rockville, MD, USA) recommended by The International Standard Organization (ISO-10993-5-2009) for testing components with potential biomedical application. Human fetal osteoblasts (hFOB 1.19, Manassas, VA, USA) were used in parallel. The L929 cell line is a standard model used for in vitro biocompatibility studies due to their stability and optimal division period, and adherent growth. L929 cells were cultured in RPMI-1640 medium (Sigma-Aldrich, St. Louis, MO, USA) supplemented with inactivated bovine serum (Cytogen, Lodz, Poland) and antibiotics: penicillin (100 U/mL) and streptomycin (100 μg/mL) in standard conditions (37 °C, 5% CO_2_, >90% humidity). hFOB 1.19 cells were cultured in DMEM/Nutrient Mixture F-12 Ham (Sigma-Aldrich) supplemented with inactivated bovine serum (Cytogen) and geneticin (0.3 mg/mL) in incubator conditions set to 34 °C, 5% CO_2_ and >90% humidity. For the test, 100 μL of cell suspension (L929: 1 × 10^5^/^mL^ and hFOB 1.19: 4 × 10^5^/^mL^) was applied to a 96-well cell culture plate and incubated overnight in appropriate incubator conditions for L929 and hFOB 1.19 to allow cells to adhere and create monolayers. Cell cultures were monitored microscopically to check confluency. Next, the tested materials (equal to 1/10 of the well area) were applied to wells containing monolayers of L929 or hFOB 1.19 cells. The cell cultures were incubated for 24 h with the tested samples and controls under the appropriate incubator conditions (L929: 37 °C and hFOB 1.19: 34 °C). After the incubation, the cells’ condition was verified microscopically, and then 20 μL of MTT (Sigma-Aldrich) (5 mg/mL) was added to each well and the mixture was incubated for 4 more hours. Following the 4 h-incubation, when formazan crystals had formed, plates were centrifuged (1200 rpm, 10 min) and supernatants were replaced with 200 μL of DMSO per well. After dissolving formazan crystals (approximately one-minute incubation at room temperature), the absorbance was measured at 570 nm (Multiskan EX, ThermoFisher, Waltham, MA, USA).

The positive control (PC, control of viability—cells in the culture medium only), negative control (NC, cytotoxicity control—cells treated with 2% saponin in order to induce cytotoxic effect), and reference certified material (R, biomaterial derived from medically certified peripheral venous catheter) controls were included in the study.

#### 3.5.2. In Vitro Immunocompatibility Assay on THP1-Blue™ NF-ĸB Monocytes

The THP1-Blue™ NF-ĸB human monocytic cell line (Invivogen, San Diego, CA, USA) was used in a screening assay determining whether the tested samples induced activation of monocytes on the in vitro level. The activation via one of the Toll-like receptors (TLR) induced the nuclear factor (NF)-κB, and subsequently the release of secreted embryonic alkaline phosphatase (SEAP) was detected in the cell culture using Quanti-Blue™ reagent (Invivogen). The assessment of immunocompatibility was performed as described previously [24]. Prior to the experiments, the monocytes were cultured in RPMI 1640 growth medium supplemented with 10% of inactivated fetal bovine serum (Cytogen, Lodz, Poland), 2 mM L-glutamine, 25 mM HEPES supplemented with penicillin (100 U/mL), streptomycin (100 µg/mL) and the selective agents normocine (100 µg/mL) and blastocidine (10 µg/mL) (Invivogen), and incubated at 37 °C in 5% CO_2_. The 96-well culture plates were inoculated with the obtained cell suspension (5 × 10^5^ cells/mL) at the working volume of 180 μL/well. Then, the tested samples were transferred (in four repeats each) into wells containing THP1-Blue™ NF-ĸB cells and incubated under standard conditions for 24 h. The wells containing cells in cell culture medium and cells treated with a non-immunogenic control biomaterial derived from medically certified peripheral venous catheter were used as negative control; cultures treated with Escherichia coli lipopolysaccharide (LPS) O55:B5 (Sigma Aldrich, Saint Louis, MO, USA) at the final concentration of 5 ng/mL were used as a positive control for monocyte activation. After incubation, the plates were centrifuged (1400 rpm, 10 min) and individual supernatants (20 μL) were transferred into the corresponding wells of a plate containing 180 μL/well of Quanti-Blue™ detection reagent and incubated for another 3 h; absorbance was determined at 650 nm wavelength (Spectra Max, Thermo Fisher, Waltham, MA, USA).

#### 3.5.3. Complement Fixation Test

A whole blood assay, including a complement-mediated immune response, was performed according to the PN-EN ISO 10993-4 norm of biological evaluation of medical devices, Part 4: Selection of tests for interaction with blood. Briefly, triplicate 5PLA_30WMA and 5PLA_30WMA_ALN composites were incubated with standardized human serum and guinea pig serum (the source of complement) for 60 min at 37 °C in a water bath. Next, a test indicator—4% SRBC (sheep red blood cells)—pre-bound to anti-SRBC antibodies (amboceptor) was added and incubated for 30 min at 37 °C in the water bath. After that time, lysis of erythrocytes was observed. Samples containing guinea pig serum and SRBC pre-bound to amboceptor served as the positive control (100% of hemolysis), whereas PBS and SRBC pre-bound to amboceptors served as the negative control (no lysis).

#### 3.5.4. Visualization of Cell Adhesion

In order to perform the visualization of the cell adhesion, composites (5PLA_30WMA and 5PLA_30WMA_ALN) were placed in an individual well of the non-adherent Nunclon Delta Surface 24-well culture plate (Nunc, Thermo Fisher Scientific, Waltham, MA, USA) in four replicates, each under aseptic conditions. Firstly, a 1 h incubation was performed (1 mL/well of culture medium at 34 °C and 5% CO_2_) in order for the composites to absorb the liquid and fill their porous compartments with it. Afterwards, the composites were transferred to a new non-adherent 24-well plate, and 1 mL of hFOB 1.19 osteoblast suspension (5 × 10^5^ cells/mL) was added to each well and incubated for 24 h 34 °C and 5% CO_2_. After incubation, the composites were removed and washed gently with phosphate buffered saline (PBS), and the cells were fixed with 3.7% paraformaldehyde (Sigma-Aldrich, Saint Louis, MO, USA) for 20 min at RT. The permeabilization was performed using 0.1% Triton X-100 in PBS for 15 min at RT. The cells were stained with fluorescent dyes (Texas Red^®^-X and DAPI, Thermo Fisher Scientific, Waltham, MA, USA) enabling their visualization by confocal microscopy as described before by Szustakiewicz et al. [72]. Composites were observed in a confocal macroscope (Leica TCS LSI, Leica Microsystems, Frankfurt, Germany) using a 5×/0.50 LWD objective and imaged with the following fluorescence conditions: Texas Red^®^-X (λ_Ex_ 596 nm/λ_Em_ 615 nm) and DAPI (λ_Ex_ 360 nm/λ_Em_ 460 nm). The Leica Application Suite X software (LAS X, Leica Microsystems, Frankfurt, Germany) was used for cell imaging. The confocal analysis was performed in the Laboratory of Microscopic Imaging and Specialized Biological Techniques at the Faculty of Biology and Environmental Protection at the University of Lodz.

#### 3.5.5. Quantification of the Osteoinductivity Markers

**Figure 17:**
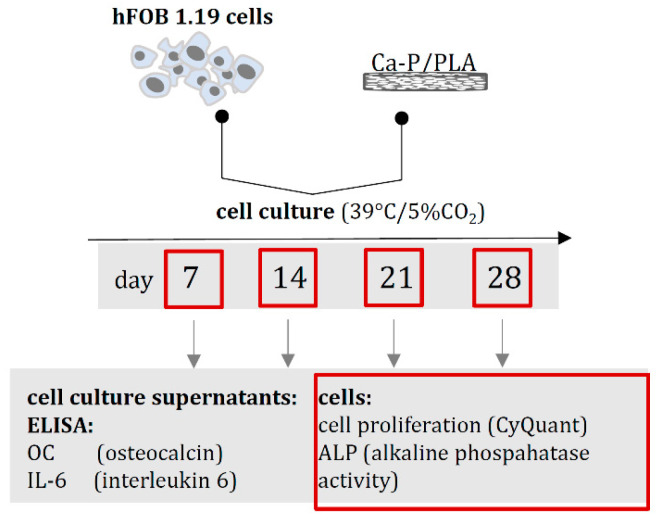
The timeline for biological studies of Ca-P/PLA composites based on quantification of osteoinduction markers: OC (osteocalcin), IL-6 (interleukin)-6, cell proliferation and alkaline phosphatase (ALP) activity evaluated at selected time points of 28-day cell culture.

The osteoblasts hFOB 1.19 cell suspensions were cultured and prepared prior to the experiments as previously described (Figure 17) [72,73]. Briefly, Ca-P/PLA composites were placed into 24-well culture plates (Nunclon Sphera, Nunc, Thermo Fisher Scientific, Waltham, MA, USA) and soaked with 200 µL of osteogenic medium (DMEM/F12 medium supplemented with 1% inactivated fetal bovine serum (HyClone, Cytiva, Marlborough, MA, USA), containing 0.3 mg/mL geneticin, 50 μg/mL ascorbate-2-phosphate (Sigma-Aldrich, Saint Louis, MO, USA), 1 μM dexamethasone (Sigma-Aldrich, Saint Louis, MO, USA), and 10 mM β-glycerophosphate (Sigma-Aldrich, Saint Louis, MO, USA) and incubated for 2 hrs at 39 °C and 5% CO2. Then osteoblasts (5 × 105 cells in 20 µL) were seeded on the top of each Ca-P/PLA composite and were cultured at 39 °C for 28 days. The growth medium was partly replaced with a fresh medium every 3–4 days, and supernatants were collected on days 7, 14, 21 and 28 of incubation, and were stored at −80 °C until further evaluation of the soluble markers: see Evaluation of the OC and IL-6 release profile. Cell lysates collected on days 7, 14, 21 and 28 were used for proliferation analysis: see Osteoblast quantification within the Ca-P/PLA scaffold. For ALP quantification, see Activity of the alkaline phosphate.

#### 3.5.6. Osteoblast Quantification within the Ca-P/PLA Scaffold

The number of osteoblasts at each timepoint of cell culture was evaluated on the basis of DNA content measured using a CyQUANT^®^ Cell Proliferation Assay Kit (Invitrogen, Thermo Fisher Scientific, Waltham, MA, USA) as described previously [73]. The osteoblasts exposed to Ca-P/PLA scaffolds were treated as indicated above (see Quantification of the osteoinductivity markers), and the experiment was carried out in triplicate and with two technical repeats. On days 7, 14, 21 and 28, scaffolds colonized with cells were washed with PBS and frozen at −80 °C. Prior to the assay, samples were thawed at room temperature and lysed in CyQuant-GR dye buffer. The standard curve based on cells in different cell densities was used to convert sample fluorescent units into cell number per scaffold. Fluorescence was measured at an emission wavelength of 520 nm and excitation wavelength of 480 nm on SpectraMax^®^ i3x Multi-Mode Microplate Reader (Molecular Devices, San Jose, CA, USA).

#### 3.5.7. Alkaline Phosphate Activity

The alkaline phosphatase (ALP) activity was measured in cell lysates on the basis of p-NPP (para-nitrophenylphosphate) hydrolysis, as described previously [73]. Briefly, 100 µL p-NPP was (4 µg/µL) added to 100 µL samples on a 96-well plate and incubated at 37 °C for 30 min. Then, 2 M NaOH was added to stop the enzymatic reaction. Optical density at 405 nm was determined using the Multiskan EX reader (Thermo Scientific, Waltham, MA, USA). The experiment was carried out in triplicate and with two technical repeats. The standard curve (range from 0 IU/mL to 10 IU/mL) of ALP (Molecular Biology, Thermo Scientific, Waltham, MA, USA) was used to calculate the enzyme activity in each sample.

#### 3.5.8. Evaluation of the OC, OPN and IL-6 Release Profile

The levels of OC, OPN and IL-6 produced by cells in the culture medium were determined by the enzyme-linked immunosorbent assays (R&D Systems, Minneapolis, MN, USA) according to the manufacturer’s instructions [74]. The minimum detectable levels were 156.5 pg/mL for OC, 62.5 pg/mL for OPN and 9.38 pg/mL for IL-6. Absorbance values at 450 nm of samples and standards at serial concentrations were obtained by Multiskan EX reader (Thermo Scientific, Waltham, MA, USA) and translated into the concentration of evaluated biomarker.

#### 3.5.9. Osteoclast-Mediated Resorption Assay

Osteoclasts cells were derived from human monocytes cultured in α-MEM medium (Sigma-Aldrich, St. Louis, MO, USA) supplemented with 10% inactivated bovine serum (Cytogen, Lodz, Poland), antibiotics: penicillin (100 U/mL) and streptomycin (100 μg/mL) and RANKL (100 ng/mL; Merck KGaA, Darmstadt, Germany) and recombinant human macrophage colony stimulating factor (M-CSF; 100 ng/mL; Sigma-Aldrich, St. Louis, MO, USA) [75].

Monocytes were isolated from the blood of healthy donors from the Central Blood Bank in Lodz using a two-step gradient isolation protocol. In the first step, PMBC cells were obtained based on the Histopaque 1077 (Sigma-Aldrich, St. Louis, MO, USA) gradient isolation method in accordance with the manufacturer’s recommendations. After isolation of PBMC layer containing lymphocytes and monocytes, cells were washed three times in PBS/EDTA solution, and the pellet was resuspended in RPMI-1640 medium (Sigma-Aldrich, St. Louis, MO, USA) supplemented with inactivated bovine serum (Cytogen, Lodz, Poland) and the antibiotics penicillin (100 U/mL) and streptomycin (100 μg/mL). Cells were counted and a solution containing 5 × 10^6^ cells/mL was prepared for further Percoll^®^ (Sigma-Aldrich, St. Louis, MO, USA) gradient isolation. Isolated monocytes in the 1 mL suspension (3 × 10^6^ cells/mL) were added to each well of the 6-well culture plates (Nunclon Sphera, Nunc, Thermo Fisher Scientific, Waltham, MA, USA) and incubated for 7 days at 37 °C and 5% CO_2_ in medium supplemented with RANKL and M-CSF α-MEM. The 500 μL of medium was replaced with fresh medium on day 4 of incubation.

After incubation, osteoclast cells were detached, counted and seeded (2.5 × 10^4^ cells/slice/50 μL) on the composite (Ø~3 mm) placed in the 24-well culture plate (Nunclon Sphera, Nunc, Thermo Fisher Scientific, Waltham, MA, USA). After 30 min of pre-incubation, 500 μL of α-MEM medium (supplemented with 10% of inactivated bovine serum and the antibiotics penicillin (100 U/mL) and streptomycin (100 μg/mL) was added to each well and incubated for 5 more days in 37 °C and 5% CO_2_.

For the evaluation of the resorption pits, composites were stained with Coomassie brilliant blue and analyzed with light microscopy [75].

#### 3.5.10. Statistical Analysis

GraphPad Prism 6 (GraphPad Software, San Diego, CA, USA) was used to perform the statistical analysis. The normality of the data was verified by the Kolmogorov–Smirnov test. The Mann–Whitney U test was used for comparison between the two groups for the variables that did not present normal distribution. The experiments were carried out in at least triplicate and with two technical repeats. *p* < 0.05 was considered as statistically significant.

## 4. Conclusions

In this study, we showed that the application of the new dual modification strategy in the Ca-P/PLA composite obtained a porous biomaterial with improved physicochemical and biological properties.

- The surface modification of Ca-P whiskers as composite-reinforcing fillers was successfully performed with two different compounds (LA or APTES). It was shown that APTES adsorbed on the surface of the whiskers resulted in a greater improvement in mutual compatibility and adhesion between the composite components than LA, and thus improved the strength properties of the material more successfully.
- The second stage of modification was also carried out successfully, and consisted in adsorbing sodium alendronate, the first-line drug for osteoporosis, on the surface of a selected composite obtained with 30% addition of APTES-modified Ca-P whiskers.
- The material, developed and produced with an innovative method of dual modification, was characterized by a microstructure suitable for porous materials dedicated to bone fillings, mechanical strength at the level of a natural cancellous bone and the ability to release an active substance.
- We showed that PLA-based composites were non-cytotoxic towards standard L929 and target hFOB 1.19 cell lines, and they did not activate the NF-κB signaling pathway in human THP1-Blue™ NF-ĸB monocytes. We proved that the dual-modified composite containing alendronate increased the proliferative potential and enabled cell growth of human fetal osteoblasts (hFOB 1.19) compared to the control material lacking alendronate. Most importantly, we showed that the presence of ALN stimulated osteoblasts to differentiate, which was manifested by the significant increase in OC and ALP production. Sodium alendronate binds to hydroxyapatites in the bones, and thus inhibits the activity of osteoclasts, which cause bone tissue resorption. However, sodium alendronate does not inhibit the bone formation process. Hence, the modification of the composites with alendronate resulted in efficient scaffold colonization, which in turn would be expected to translate to high-density bone tissue formation and an enhanced regenerative potential in such composites. Overall, the biological evaluation of the 5PLA_30WMA_ALN composite shows it to be a very promising candidate suitable for future biomedical engineering applications.

## Figures and Tables

**Table 1:** Compressive properties of the porous Ca-P/PLA composites (the values were presented with standard deviation).

Sample	Compressive Modulus [MPa]	Compressive Stress (10% Strain) [MPa]	Compressive Stress (20% Strain) [MPa]	Compressive Stress (50% Strain) [MPa]
5 PLA	2.897 ± 1.451	0.212 ± 0.032	0.296 ± 0.036	0.662 ± 0.104
5 PLA_10W	2.531 ± 0.842	0.235 ± 0.030	0.331 ± 0.029	0.646 ± 0.046
5 PLA_20W	2.115 ± 0.423	0.209 ± 0.028	0.305 ± 0.034	0.626 ± 0.048
5 PLA_30W	1.921 ± 0.595	0.179 ± 0.034	0.265 ± 0.041	0.577 ± 0.054
5 PLA_50W	2.632 ± 1.397	0.199 ± 0.023	0.288 ± 0.016	0.604 ± 0.035
5 PLA_10WMA	3.312 ± 1.005	0.271 ± 0.022	0.368 ± 0.016	0.704 ± 0.018
5 PLA_20WMA	3.138 ± 1.473	0.310 ± 0.056	0.420 ± 0.040	0.760 ± 0.068
5 PLA_30WMA	3.795 ± 1.189	0.347 ± 0.057	0.438 ± 0.049	0.769 ± 0.048
5 PLA_50WMA	3.957 ± 0.374	0.340 ± 0.042	0.459 ± 0.037	0.841 ± 0.049
5 PLA_10WMLA	4.044 ± 1.499	0.295 ± 0.033	0.404 ± 0.037	0.811 ± 0.075
5 PLA_20WMLA	2.894 ± 1.100	0.240 ± 0.031	0.344 ± 0.027	0.709 ± 0.033
5 PLA_30WMLA	2.645 ± 0.963	0.238 ± 0.034	0.333 ± 0.018	0.683 ± 0.010

**Table 2:** Compressive properties of dual modified composite: 5PLA_30WMA_ALN (the values were presented with standard deviation).

Sample	Compressive Modulus [MPa]	Compressive Stress (10% Strain) [MPa]	Compressive Stress (20% Strain) [MPa]	Compressive Stress (50% Strain) [MPa]
5 PLA_30WMA_ALN	4.832 ± 1.526	0.247 ± 0.027	0.325 ± 0.024	0.653 ± 0.042

**Table 3:** Percentage composition of the composites.

Sample	The Content of Individual Components in the Composite [% wt]			
	PLA	W	WMA	WMLA
5PLA	100	-	-	-
5PLA_10W	90	10	-	-
5PLA_20W	80	20	-	-
5PLA_30W	70	30	-	-
5PLA_50W	50	50	-	-
5PLA_10WMA	90	-	10	-
5PLA_20WMA	80	-	20	-
5PLA_30WMA	70	-	30	-
5PLA_50WMA	50	-	50	-
5PLA_10WMLA	90	-	-	10
5PLA_20WMLA	80	-	-	20
5PLA_30WMLA	70	-	-	30

## Data Availability

The data generated during this study are available at ŁUKASIEWICZ Research Network Institute of Ceramics and Building Materials, Center of Ceramic and Concrete in Warsaw, Biomaterials Research Group, Postępu 9, Warsaw, 02-676, Poland, and are available from the corresponding author upon request.
